# Reducing the reliance on nitrogen fertilizer for wheat production

**DOI:** 10.1016/j.jcs.2013.12.001

**Published:** 2014-05

**Authors:** Malcolm J. Hawkesford

**Affiliations:** Plant Biology and Crop Science Department, Rothamsted Research, Harpenden, Hertfordshire, AL5 2JQ, UK

**Keywords:** Nitrogen, Cereals, Wheat, Yield, GPD, grain protein deviation, HI, harvest index, NHI, nitrogen harvest index, NUE, nitrogen use efficiency, NUpE, nitrogen uptake efficiency, NUtE, nitrogen utilization efficiency, SSA, sub-Saharan Africa, WGIN, Wheat Genetic Improvement Network

## Abstract

All crops require nitrogen (N) for the production of a photosynthetically active canopy, whose functionality will strongly influence yield. Cereal crops also require N for storage proteins in the grain, an important quality attribute. Optimal efficiency is achieved by the controlled remobilization of canopy-N to the developing grain during crop maturation. Whilst N will always be required for crop production, targeting efficient capture and use will optimise consumption of this valuable macronutrient. Efficient management of N through agronomic practice and use of appropriate germplasm are essential for sustainability of agricultural production. Both the economic demands of agriculture and the need to avoid negative environmental impacts of N-pollutants, such as nitrate in water courses or release of N-containing greenhouse gases, are important drivers to seek the most efficient use of this critical agronomic input. New cultivars optimised for traits relating to N-use efficiency rather than yield alone will be required. Targets for genetic improvement involve maximising capture, partitioning and remobilization in the canopy and to the grain, and yield *per se*. Whilst there is existing genetic diversity amongst modern cultivars, substantial improvements may require exploitation of a wider germplasm pool, utilizing land races and ancestral germplasm.

## Highlights

• Trends in yields, NUE and N fertilizer application are described.
• NUE terms are explained.
• Specific traits for improvement are listed.
• Existing variation is outlined.
• An overview for the scope for improvement is presented.

## 1 Introduction

There is an absolute requirement for N for plant growth, and crop yields and quality depend upon substantial N inputs. Chemical N fertilizers were first used in agriculture in the 19th century, and subsequently to a much greater extent after the development of the Haber–Bosch process at the beginning of the 20th century. At the present time, more than half of the chemically fixed N is used by agriculture, amounting to in excess of 80 Mt per year, worldwide.

Cereal crops are a major staple food worldwide, contributing more than 50% of total human calorie input directly. Crop production needs to continue to grow with increasing demand, and both improved yields and sustainability are major challenges facing current agriculture. Worldwide production systems vary greatly depending on climatic and soil fertility factors. In all agricultural systems there is a need for adequate nutrients, usually supplied as fertilizer in areas of higher production. N is a major macronutrient often limiting plant growth. The application of N fertilizers in agriculture has increased markedly since the middle of the 20th century due to the impact of the ‘green revolution’ which combined best agronomic practice with the use of germplasm better able to respond to applied N. Increasing N supply to a crop drives the production of a greater canopy biomass with the potential for higher photosynthesis and productivity. However, a penalty for a large biomass can be a susceptibility to lodging. The adoption of short and stiff strawed cultivars substantially overcomes this issue, which may be further alleviated with the use of chemical growth regulators. In addition, the high harvest indexes (ratio of grain to total biomass at harvest) associated with short cultivars, further contributes to resource use efficiency, with little residual N remaining in the straw after grain harvest.

Most measures of NUE (nitrogen use efficiency) relate production as a function of inputs, and given constant inputs, any yield increase will be reflected in greater NUE. However, comparisons of high versus low input systems are more difficult with such crude definitions, giving misleading indications of high efficiency at low or zero inputs.

Although greater N application has produced higher yields, this is not a linear relationship (see below) and there is an economic optimum application offsetting incremental yield increase against the cost of additional N inputs, which needs to be determined for individual cultivars (Foulkes et al., 1998; King et al., 2003). Availability of N has impacts throughout crop development, affecting seedling establishment, tillering, canopy development as well as grain filling, all of which have the potential to influence final yield and together determine the N requirements of the crop. The optimization of crop production and NUE is a complex problem and will require a complex set of solutions to achieve improvement.

## 2 Trends in yield and NUE

**Fig. 1:**
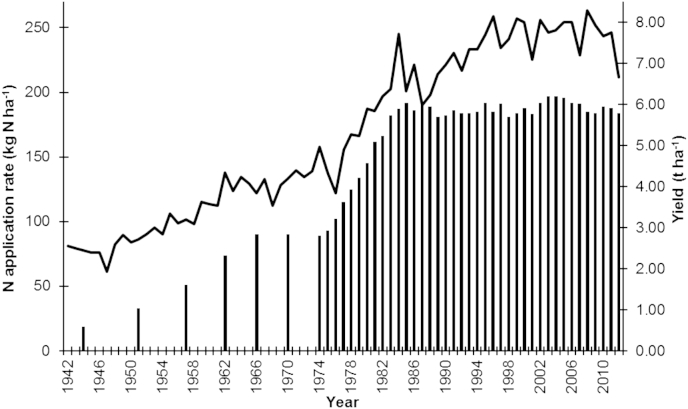
Wheat yields (continuous line) since 1942 in Great Britain and available information on the pattern of N application rates for England and Wales (bar chart) to cereal crops over the same period. Data extracted from UK Department of Food and Rural Affairs, the Rothamsted archive and British Survey of Fertilizer Practice. Figure courtesy of Chris Dawson and Associates, UK.

In the second half of the 20th century cereal yields have increased, for example for wheat, worldwide from 1 to 3 t ha^−1^ (Fischer and Edmeades, 2010; Hawkesford et al., 2013) and in the UK from less than 3 t ha^−1^ to around 8 t ha^−1^ (Fig. 1). This is exemplified by data on UK wheat yields (Fig. 1). Increases were greatest in the 1970s due to the introduction of short straw cultivars which enabled higher N inputs, facilitating larger canopies with reduced susceptibility to lodging. Since then, yield rises have been more modest or have even stagnated both in the UK and elsewhere (Brisson et al., 2010). In the UK, N fertilizer inputs increased up to the 1980s, supporting the increasing yields. Since then, legislation has limited N application and UK average N fertilizer rates have stabilised at under 200 kg N ha^−1^ (Fig. 1). The relatively modest recent yield increases (1–2 t ha^−1^ over the past 30 years) with stable N inputs equate to a higher NUE at the national level in the UK.

**Fig. 2:**
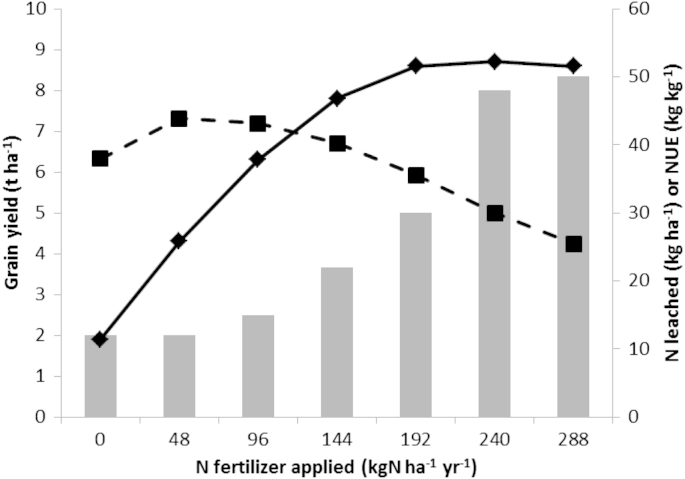
Illustration of impact of N fertilizer application on winter wheat yield (solid line, diamonds), N-losses due to leaching (bar chart) and estimated grain NUE (dashed line, squares). Data taken from the Broadbalk long-term experiment at Rothamsted, from 1990 to 1998 (cv. Apollo 1990–1995 and cv. Hereward 1996–1998). Modified from Hawkesford (2011) and used with permission (Wiley and Sons, Ltd: Chichester).

**Fig. 3:**
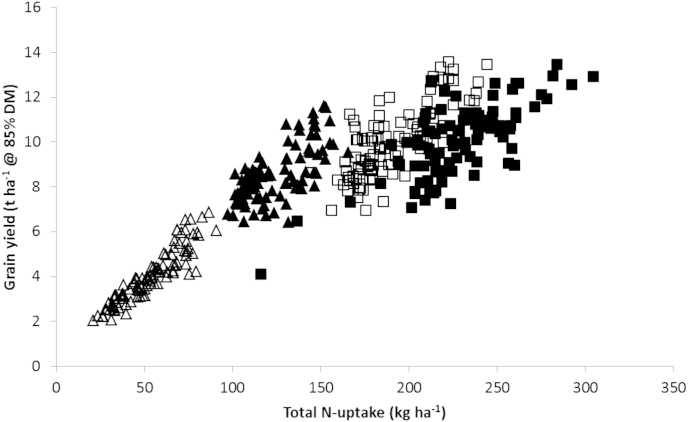
Impact of N fertilizer application on grain yield and total N taken up by the crop at maturity. Data are from the Defra Wheat Genetic Improvement Network trials (2007–2010), analysed in accordance with Barraclough et al. (2010). Data are available on the WGIN website (http://www.wgin.org.uk/). N application rates are 0, 100, 200 and 350 kg N ha^−1^ (open triangles, closed triangles, open squares, closed squares, respectively).

The impacts of adding more N are illustrated in Figs. 2 and 3. Data taken from the Broadbalk classical experiment at Rothamsted (Fig. 2) illustrate the positive benefit of increased yield with increasing N fertilizer addition up to around 192 kg ha^−1^, after which there is little apparent increase in yield for the cultivars tested. These data also illustrate the negative impact of increased leaching at the higher N applications. When NUE is calculated as a function of grain yield per estimated N input, this decreases with the increasing N input (Hawkesford, 2011).

The positive impact of increasing yield together with the additional benefit of increasing N content of the crop with increasing N application is shown from an analysis of experimental data from the UK Wheat Genetic Improvement Network (WGIN) in trials at Rothamsted (Fig. 3). With N fertilizer application between 0 and 200 kg N ha^−1^, both yield and N uptake increase substantially. At the highest N application rate (350 kg ha^−1^), no further yield increase occurs although further N uptake is apparent. Much of the additional N taken up is manifest in higher grain N content (data not shown). The scatter at each N input rate reflects the wide variation in cultivars used in the trials and the contrasting weather patterns in the 4 years of the trials presented. The inability of the crop to respond to the increased N above 200 kg ha^−1^ in terms of increased yield reflects factors other than N-limited yield, most likely source productivity. This source limitation may be intrinsic photosynthetic efficiency or water limitation. The genetic potential of these cultivars should be well in excess of the mean achieved under these treatments (10–11 t ha^−1^). The only modest increase in grain N, in spite of a huge increase in N application (350 compared to 200 kg N ha^−1^), indicates either poor capture or a lack of sinks to utilize the available N.

## 3 Definitions and nitrogen cycles

Reducing the N requirements of cereals implies an increase in efficiency of use of applied N. Greater yields with less inputs would seem to be an ideal trait, however, there are severe constraints on such a simplistic goal and it is necessary to consider individually the final crop product and the component physiological traits which contribute to NUE. Increasing yield with no increase in inputs will by definition give greater use efficiency, but this may be at the expense of quality attributes. These issues are discussed below.

There are many definitions of NUE (Fageria et al., 2008; Good et al., 2004). For example, NUE may be defined as yield per unit of N available to the crop (Moll et al., 1982). Available N includes fertilizer inputs, atmospheric deposition and mineralization within the soil. N available from soil mineralization is dependent upon soil organic matter and the history of the crop land use. Additionally, rotations including leguminous crops will contribute to soil N from biological N fixation.

The overall trait of NUE may be divided into N uptake efficiency (NUpE) and N utilization efficiency (NUtE), with NUE being the product of the two (Moll et al., 1982). NUpE may be defined as the amount of N taken up by the crop as a fraction of the amount available to the crop from all sources. This trait is predominantly associated with root structure and functioning, although available sinks may limit the ability to efficiently take up available N. Ideal traits will include early root proliferation to scavenge N before fertilizer application, proliferation near to the surface to enable capture of applied N, and later longer roots with proliferation at depth to access deeper N reserves and leached N. Fertilizer use efficiency is a variant on this trait and refers specifically to the percentage recovery of applied fertilizers and is usually determined by tracer studies. Overall, NUE on a worldwide scale has been estimated to be as low as 33% (here defined as [total grain N removed − N coming from soil]/fertilizer N applied) for all cereals combined (Raun and Johnson, 1999).

NUtE for grain is defined as grain yield divided by the amount of N taken up. Physiologically this is due to the photosynthetic efficiency of the canopy and the ability to produce grain yield as a function of the photosynthate fixed. Highest NUtE will be obtained if N uptake is kept to a minimum. As already indicated, whilst initially N is required for the production of the photosynthetic machinery in the canopy, it is ultimately also required to support the integrity of the grain, and additionally for the synthesis of storage proteins, contributing directly to quality (Shewry, 2007).

**Fig. 4:**
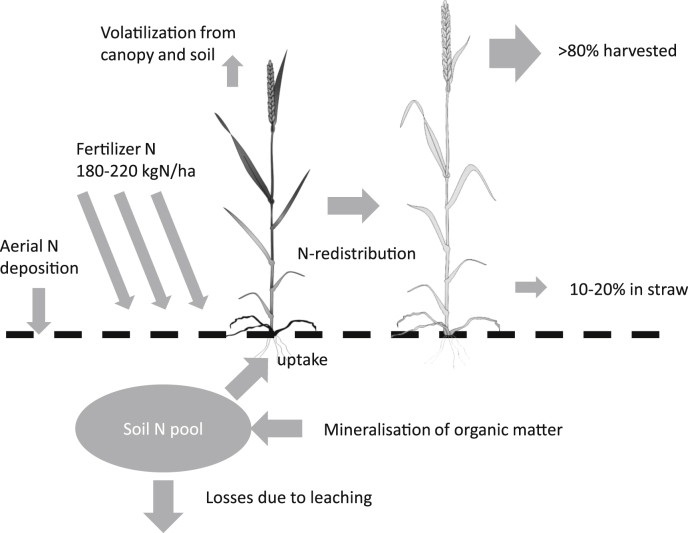
Idealized major fluxes of N in a high yielding wheat crop. Fertilizer application of 180–200 kg/ha is representative of the UK and is likely to be applied in a 3-way split. Width of arrow is a qualitative indication of size of flux.

The crop N cycle is summarized in Fig. 4. This cartoon summarises the major inputs, outputs and intermediate fluxes as might be expected in a highly productive intensive wheat production system. N inputs are predominantly from fertilizer applications, optimally supplied in multiple doses, timed to supply the N needs of the crop at different developmental points. Additional N for the crop is provided by aerial deposition and mineralization of organic matter in the soil, processes that occur throughout the year and provide important resources prior to the first fertilizer application. Ideally, losses due to leaching will be minimized if fertilizer application quantities and timings accurately match crop needs. Prior to grain filling, N is required for canopy establishment and also for root production. Allocation to root proliferation rather than the shoot may be under nutritional control. After flowering and during grain filling, any further N taken up is likely to be allocated directly to the grain; however, much of the grain N will be from re-distribution from the canopy, thus ensuring an overall optimal usage of N taken up. The ratio of N in the grain as a function of the total N taken up is the N harvest index (NHI); amongst a diverse germplasm set, NHI was estimated to be more than 80% and was relatively insensitive to the N fertilizer supply (Barraclough et al., 2010).

In the UK, NUE may be calculated as being between 30 and 40 kg grain per kg N available (Fig. 2); for a 10 t ha^−1^ crop (10,000 kg) taking up 250 kg N (200 kg N as fertilizer; 80% of biomass and N in grain) this value would be 40 based on the Moll et al. (1982) definition or 75% on the Raun and Johnson (1999) definition.

In contrast, in many areas of sub-Saharan Africa (SSA) where often little fertilizer N is applied, the calculated NUE may exceed 100%, using the NUE definition defined by Raun and Johnson (1999). Applying little or no fertilizer but harvesting of crops leads to depletion of soil mineral reserves and deterioration of soil quality. Yields in such circumstances are clearly not sustainable and the application of NUE estimations is erroneous. Critically, in the absence of fertilization, nutrient mining (of N and all other essential nutrients) will result in even less productive land, a major issue in, for example, SSA (Edmonds et al., 2009); in this case, crop improvement is best addressed by minimizing nutrient removal or focussing on recycling. However, the situation in SSA is rapidly changing, with adoption of fertilizers by many farmers, albeit still at relatively low levels, aided by state subsidies (Druilhe and Barreiro-Hurlé, 2012).

## 4 Specific traits for potential improvement

### 4.1 Identifying novel genes

One approach gaining in popularity for discovering genes underpinning variation in complex traits such as NUE or responses to available N is via transcriptome analysis. This has been applied to analyze specific steps such as senescence (Gregersen and Holm, 2007; Howarth et al., 2008) or grain filling (Hansen et al., 2009; Wan et al., 2008) in cereals, and in roots and shoots in relation to nitrate supply for model plants such as tomato (Wang et al., 2001) or Arabidopsis (Wang et al., 2003). Large numbers of responsive genes have been identified, however, specific attribution of identified genes to determining traits of interest has not been generally successful; more sophisticated genotype–environment–trait correlations will be required to narrow down candidate genes. Generally, more effort has focussed on exploiting pre-existing physiological and biochemical knowledge of N assimilation and targeting germplasm diversity of generating germplasm with modified expression of potential targets.

### 4.2 Targeting specific genes and processes

Specific key genes have been targeted as potential routes for the improvement of NUE (McAllister et al., 2012). Most candidate genes targeted for genetic improvement code for components of either the N uptake or N assimilatory pathways which have been suggested to be rate-limiting steps. Target genes are not always obvious: in one case, a modified expression of alanine amino transferase was reported to have a marked effect on plant NUE (Good et al., 2007; Shrawat et al., 2008), however, the exact mechanism for the impact on NUE remains elusive. Alanine amino transferase catalyses the reversible transfer of an amino group from glutamate to pyruvate to form 2-oxoglutatarate and alanine. The overexpression is only effective when targeted at the root epidermis using a tissue specific promoter and it is possible that this reaction helps create a local sink for N taken up and relieves some feedback inhibition. In addition there appears to be a positive impact on root proliferation which would enhance N capture (Shrawat et al., 2008). Whatever the mechanism, a substantial positive impact on yield at sub-optimum N inputs has been reported (Good et al., 2007).

### 4.3 N uptake

Efficient N utilization has to begin with efficient capture. This is primarily a root trait and is dependent upon both root architecture and root functioning. N is taken up via the roots mainly in the form of nitrate in most agricultural soils, but also as ammonium and to a lesser extent as organic N in the form of amino acids. Uptake and transport across cell membranes for each of these forms is catalyzed by one or more large gene families, and transport systems are highly evolved, having high substrate affinity and with expression patterns often under nutritional control (Garnett et al., 2013; Williams and Miller, 2001). Two families of nitrate transporters have been characterized, the low affinity type and the high affinity type (see for example Daniel-Vedele et al., 1998; Williams and Miller, 2001). Both transcriptional and post-translational regulatory mechanisms contribute towards optimizing functional expression of the individual transporters and coupling expression to plant supply and demand. Most published studies have focussed on model systems such as Arabidopsis, however a recent study on maize quantified expression of both high and low affinity systems over the whole life-cycle and demonstrated how specific expression is coupled to demand (Garnett et al., 2013). Root function needs to be optimum throughout the crop cycle, from establishment through to maturity with an important role of post-anthesis N uptake in wheat contributing to crop quality (Kichey et al., 2007). However, the highly complex and efficient uptake mechanisms which exist in crop plants probably offer little opportunity for selection for higher performance.

Opportunities for breeding for improved capture may exist at the macroscopic scale in terms of root architecture and proliferation. Research on roots and variation in architecture is difficult in field situations and therefore has mostly been undertaken with laboratory studies, using various artificial systems. Hydroponic, rhizotron and soil column methods can give contrasting results (Wojciechowski et al., 2009). In some laboratory screens of genotypic performance at the seedling stage, traits have matched field characteristics, with measures of root proliferation (laboratory) and expression of shoot height (field) corresponding (Bai et al., 2013). The link between crop height with the adoption of dwarfing (rht) genes and the negative impact on root proliferation suggests a negative selection for this important N capture trait (Bai et al., 2013; Gooding et al., 2012). Therefore there must be scope to improve root proliferation in modern short-strawed cultivars.

Good proxy measurements for root function in the field may offer useful screening mechanisms. The most obvious field-based measurement is of total N taken up as a function of available N (NUpE), however, this is only partly a function of yield, as seen in Fig 3 (Barraclough et al., 2010). As described above, ideotypes for efficient root systems include proliferation near to the surface and at depth (Foulkes et al., 2009). The longer growing period for winter wheat allows for deeper roots to be formed, important to prevent winter leaching losses of N (Thorup-Kristensen et al., 2009), however genetic variation in either winter or spring cultivars has been barely explored.

### 4.4 Assimilation

The first steps of the assimilatory pathway are the reduction of nitrate to nitrite catalyzed by nitrate reductase and nitrite reductase. The ammonium produced is assimilated further via the glutamine synthetase (GS)/glutamate synthase (GOGAT) cycle. Notably, multiple isoforms of glutamine synthetase exist with substantial tissue-specific expression (Bernard et al., 2008; Swarbreck et al., 2011), indicative of key multifunctional roles. One important role for glutamine synthetase in photosynthetic tissue is in recapture of ammonia released during photorespiration or senescence processes (Mattsson and Schjoerring, 1996; Swarbreck et al., 2011). There may be scope for the identification of genetic diversity of expression patterns of this gene family.

Transgenic manipulation of many of the steps of the assimilatory pathway have been demonstrated to have positive effects in pot-based greenhouse experiments but few have been tested as yet in the field (Good et al., 2004; McAllister et al., 2012).

### 4.5 Photosynthetic efficiency

Improving photosynthetic efficiency has the potential to increase yield or reduce inputs and therefore may have a major influence on NUE. A more efficient canopy requiring less N for construction but still having the same carbon fixation capacity will give increased NUE. Routes to improving photosynthetic efficiency may be through exploiting natural variation of the processes involved, or by manipulation of the biochemical pathways directly, targeting for example RuBP regeneration or catalytic properties of Rubisco (Parry et al., 2007; Reynolds et al., 2009). Alternative ideas involve improving carbon dioxide capture via concentrating mechanisms such as the introduction of C4 metabolism into C3 grasses or the exploitation of cyanobacterial carbon dioxide concentrating mechanisms (Parry and Hawkesford, 2010).

### 4.6 NHI, remobilization and stay-green phenotypes

As already stated, N is required for establishment and construction of the canopy, and specifically for the photosynthetic apparatus. However, an essential component of crop NUE is the re-use of this canopy-N for the synthesis of storage proteins in the grain during grain filling, which occurs as the crop matures. Senescence of the canopy limits further photosynthetic activity and yield generation but is essential for the remobilization of N and other minerals, which are required for optimal grain production. A larger canopy, containing more N and minerals, which has a pattern of phased senescence will provide an optimum compromise between continued photosynthesis, adequate provision of N to the grain and an overall maximum NUE.

Remobilization of N depends on both environmental and genotypic factors. Environmental factors include N fertilization (delays onset of senescence and increases amount of N for remobilization), disease pressure and drought conditions (both enhancing senescence and decreasing NUE) (Barbottin et al., 2005). There is also considerable genotypic variation in flowering and maturation time, as well as senescence kinetics.

Whilst stay-green phenotypes will prolong photosynthesis with possible beneficial effects on yield, delayed or no senescence will have negative consequences for nutrient remobilization. A NAC (NAM/ATAF1,2/CUC2-like) transcription factor (NAM-B1) present in ancestral emmer but not modern durum or hexaploid wheats was responsible for a highly efficient N and other mineral remobilization from the canopy to the grain, and was also associated with more rapid senescence (Uauy et al., 2006). This, however, have negative consequences for yield.

Delayed maturity or functional stay-green phenotypes should increase the grain filling period and boost yield. Stay-green mutants for durum wheat have been reported which show increased grain weights, total yield and improved N uptake, at least in the glasshouse conditions used in the study (Spano et al., 2003). A similar study with a bread wheat comparing fast and slow senescing lines with similar anthesis date clearly indicated that increasing the rate of senescence had a marked negative impact on yield but extending the grain filling period had little or no impact on yield and N content (Derkx et al., 2012).

### 4.7 Grain N

Whilst delivery and partitioning are viable targets for improvement, the greatest impact on reducing the demand for N fertilizers would be to reduce grain N requirements, although this clearly would be at the expense of grain protein. High protein content is of nutritional benefit, particularly if coupled with the presence of a high content of essential amino acids (Shewry, 2007); additionally, high protein is required for optimum end-use quality, for example a minimum protein content is required for bread making. Increasing N fertilizer increases grain %N (Fig. 3), total protein content, gluten content required for the viscoelastic matrix and also the proportion of gliadin proteins (Godfrey et al., 2010; Wieser and Seilmeier, 1998). Some gliadin proteins are more responsive to N supply than others (Wan et al., 2013). Both the total protein and the specific composition, particularly of high molecular weight proteins contributing to the gluten matrix contribute to critical dough properties such as dough extension. To avoid excessive N fertilizer application to achieve these qualities, either optimized protein composition or the use of bread making procedures requiring less protein are required; research into both of these areas is necessary (Shewry, 2009).

### 4.8 Grain protein deviation

**Fig. 5:**
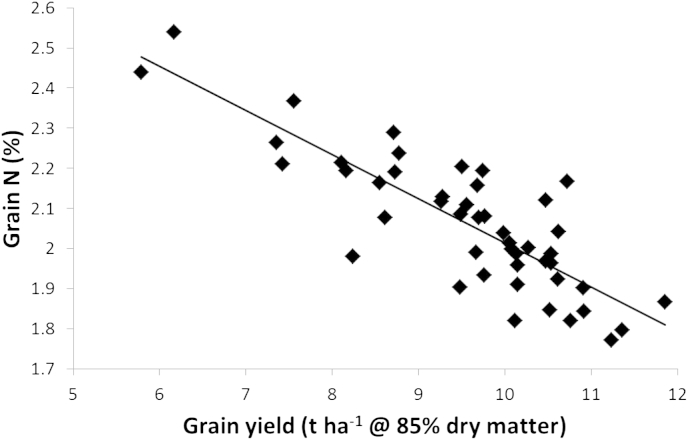
Grain protein deviation. Grain N content and grain yields for 47 cultivars, mean data over a 9-year period (2004–2012) in the WGIN trials at Rothamsted. Analysis performed as previously described (Barraclough et al., 2010) with data published on the WGIN website (http://www.wgin.org.uk/).

Across most cultivars, an inverse relationship between yield and grain protein is apparent (Simmonds, 1995). Therefore an inevitable consequence of increased yields appears to be decreased grain protein concentration, at least under constant N supply. With a finite N supply and if HI and N remobilization are similar, increasing yield achieved by increased carbohydrate production inevitably will have the effect of diluting protein in the grain. This relationship is illustrated in Fig. 5. A few cultivars show a positive deviation (grain protein deviation; GPD) from this relationship (Monaghan et al., 2001). Breeders have selected primarily for increased yields but also for high protein content; therefore modern cultivars might be expected to have greater GPD. In fact GPD is generally modest and not a consistently expressed trait (Hawkesford and Shewry, unpublished observations).

The elucidation of characteristics which contribute to GPD remains a priority. Simply increasing N supply does not have the effect of maintaining N content, indicating some limitation in uptake, partitioning or protein synthesis. One key trait appears to be post-anthesis N acquisition (Bogard et al., 2010; Kichey et al., 2007; Monaghan et al., 2001) with anthesis date rather than rate of senescence being the most important factor (Bogard et al., 2011). Uptake will be dependent upon N availability and soil moisture along with root related traits. In many climates, the dry conditions associated with the period of crop maturation may limit post-anthesis N uptake. Generally however, the majority of grain N originates from remobilization from the canopy (Barneix, 2007), rather than from post-anthesis uptake, and mechanisms to enhance temporary accumulation in the canopy followed by effective remobilization should not be overlooked. Alternatively a high sink strength (that is high yield) may help enhance N uptake in some cases (Mi et al., 2000).

## 5 Selecting for natural variation in NUE

Whilst considerable genetic variation is apparent amongst wheat cultivars, much of this may be directly attributed to yield and HI attributes. Increases in grain yield equate with increased NUE, and increased HI results in higher yields as well as a greater proportion of N taken off in the grain. Simple comparisons of modern cultivars with older germplasm, and particularly land races, using many of the standard measures of NUE is difficult due to the poor HI and low yields of older material. Older cultivars, land races or ancestral relatives may have high biomass potential or be effective at N-scavenging and uptake. Appropriate screens for specific traits, particularly amongst the more exotic germplasm are essential.

### 5.1 How much variation?

Several studies have quantified genetic variation in NUE parameters in modern cereals (Barraclough et al., 2010; Bingham et al., 2012; Foulkes et al., 1998; Gaju et al., 2011; Ortiz-Monasterio et al., 1997; Sadras and Lawson, 2013; Sylvester-Bradley and Kindred, 2009). Progress in NUE amongst historic CIMMYT (Centro Internacional de Mejoramiento de Maíz y Trigo) cultivars (10 cultivars released between 1950 and 1985) indicated progress in both NUpE and NUtE; furthermore NUpE was the major contributor at low N and NUtE at higher N (Ortiz-Monasterio et al., 1997). Substantial independent variation in NUp and NUtE was observed in a study of 39 UK and European modern wheats, over 4 seasons grown at different N inputs (Barraclough et al., 2010). Similarly in spring barley, a comparison of cultivars spanning 75 years of breeding indicated increased NUtE with increasing yield, however not necessarily matched by increased NUpE, particularly for pre-anthesis dry matter accumulation. This indicated that N uptake, at least prior to anthesis may be a very specific target for genetic improvement (Bingham et al., 2012).

Variation in NUE was observed in 14 French and UK cultivars, comparing low and high N input conditions (Gaju et al., 2011). In contrast to Ortiz-Monasterio et al. (1997), at low N supply, greatest variability was seen for NUtE rather than NUpE, and timing of onset of senescence was the most influential factor particularly at low N supply. Interestingly, timing of onset of senescence at low and high inputs did not correlate well, indicating a clear interaction with N supply; this emphasizes the need to evaluate NUE at different N inputs. Delaying senescence enhanced yield but was associated with a decreased efficiency of N remobilization.

A study of 27 UK cultivars released between 1969 and 1988 in multi-site trials at zero and optimum N application demonstrated that improved yields required increased N inputs to maintain grain N (Foulkes et al., 1998). In the UK, increased N application at the national level was not seen over this period indicating a sub-optimal application (see also Fig. 1). Foulkes et al. (1998) differentiated between NUp from soil pools (at zero N input) and fertilizer N uptake, and observed that the more modern, higher yielding cultivars were poorer specifically for the soil N-pool acquisition; the implication is that the modern cultivars although perfectly able to take up surface applied fertilizer N, have poorer root systems less able to scavenge deeper pools of N.

Under Australian conditions using cultivars released between 1958 and 2007 with a trend of increasing yield, yield per unit N remained largely unchanged due to increased N uptake. In spite of an increased NHI there was still a trend of decreasing grain %N (Sadras and Lawson, 2013).

Barraclough et al. (2010) showed that season and N input had the greatest effect on all NUE parameters measured; however, additional significant varietal variation was apparent. It is probable that analysis of a wider germplasm pool would be more fruitful for the discovery of efficient NUE cultivars. This will be most apparent when considering NUpE, with wild relatives and land races potentially having efficient scavenging mechanisms. It may be that modern cultivars selected at high N inputs may have lost some of these valuable traits. To address this issue, a comparison of selection at low input, high input and alternating between high and low N inputs was undertaken for a NUE segregating population (Van Ginkel et al., 2001). Selection for yield was best under alternating high and low N conditions or under continuous high N conditions. Critically, selection at low N did not improve selection for N uptake efficiency at low available N. This result, if broadly applicable, has substantial implications for breeding strategies, and suggests that most breeding programmes conducted at high inputs may be optimal for NUE breeding after all. This is in contrast to the conclusions arising from studies such as Foulkes et al. (1998) and Gaju et al. (2011) (see above).

It is clear that both NUpE and NUtE need to be selected independently, and that selection should be conducted at low and high N inputs to obtain greater trait differentiation. Increasing yield at the expense of grain %N will always be an issue and there is a case for determining optimal N inputs for all new cultivars rather than the default standard N application. As already stated, the selection for high NUtE amongst land races is difficult as most have low grain yields and low HI; selection for biomass is a combination of the NUp trait and high photosynthetic efficiency, and so worthy of pursuit.

## 6 Managing N in the agricultural environment

### 6.1 Agronomic practice

As already stated, worldwide, NUE varies greatly (Raun and Johnson, 1999). Fertilizer application and practise are locally adapted and account for much of the variation in NUE. Whilst there is scope for improvement, generally economics and local customs dictate common practice. Where feasible, matching application to demand, both temporally and spatially is the optimum approach. In addition, a balanced nutrition is essential for both, yield and quality, as well as for individual nutrient use efficiencies. This has been extensively documented for N and sulphur, particularly relating to wheat production (Zhao et al., 1999).

N may be applied in various forms to crop systems, ranging from organic manures through to various high analysis inorganic ammonium and nitrate salts, urea, and anhydrous ammonia. Critically the management practices for the mode and timing of application should minimize volatile or leaching losses (Matson et al., 1998). In most commercial production systems, agronomic practices are fine-tuned to maximize the economics of N uptake, however further improvements may be possible. Multiple applications, timed to coincide with critical growth periods or specifically to supplement grain N content or yield mapping for spatial optimization of application are already being adopted and may have further potential. For maximum benefit, all of these approaches need to be adopted in conjunction with optimized germplasm.

### 6.2 Nitrification inhibitors

Nitrification is the conversion of ammonia into nitrate, the latter having a higher propensity for leaching from agricultural soils. Much of the N applied worldwide is in the form of ammonium and slowing the conversion to nitrate may facilitate more efficient capture. Ammonium may be a better source of N for the plant as less energy is required for assimilation. Chemical nitrification inhibitors are available but are costly. Some plant species produce exudates which are nitrification inhibitors and the transfer of this trait more widely has been proposed as a mean of improving NUE (Subbarao et al., 2013). The potential energy costs to the plant in relation to different agronomic systems may make this an attractive solution in some but not all farming systems.

## 7 Overview: what scope is there for further improvement?

Increasing yield without increasing N inputs will lead to better NUE and an effective decrease in N requirements. However, this will be at the expense of quality. A clear way forward would be to consider all cereals, including wheat, as carbohydrate (calorie) sources and not protein crops. This does have wider nutritional and cultural issues and would impact on many of the specialist food products derived from cereal flour.

Specifically targeting capture, at all stages of crop development has scope for increasing N-use and has the benefit of reducing losses to the environment. A corollary is the need to avoid soil deterioration by effectively unsustainable mining of nutrients. There is probably only minimal scope for further improving allocation to the grain, unless there is a tendency to taller dwarf cultivars which may have better resource (light, water, minerals) capture efficiency than shorter dwarf cultivars.

The two principal components of NUE, uptake and utilization, are quite distinct processes, and not surprisingly therefore, performance in the two traits is not related; consequently they may be independently selected with a clear potential for significant gains in overall NUE (Barraclough et al., 2010; Bingham et al., 2012).

A major achievement would be the introduction of N fixation into cereals. This may be achieved by introducing symbiosis association as nodules as found in legumes, creation of endophytic nodule-independent bacterial associations or by the introduction of the entire nitrogen fixation pathway into the crop itself, perhaps targeting an organelle (Beatty and Good, 2011). All of these solutions are long-term prospects, but are being actively pursued at the present time.

Several specific steps in uptake and assimilation have been identified as potential targets for transgenic approaches, and trial transgenic germplasm has been produced although few have been field-tested. Adoption of such genetically modified material is both technically difficult and widely socially unacceptable, and is therefore only a long-term prospect. Analysis of natural variation, either at the whole trait level or of individually identified steps remains the best immediate route to crop improvement for NUE.
